# FUNCTIONAL CELL PHENOTYPE INDUCTION WITH TGF-β1 AND COLLAGEN-POLYURETHANE SCAFFOLD FOR ANNULUS FIBROSUS RUPTURE REPAIR

**DOI:** 10.22203/eCM.v039a01

**Published:** 2020-01-03

**Authors:** J. Du, R.G. Long, T. Nakai, D. Sakai, L.M. Benneker, G. Zhou, B. Li, D. Eglin, J.C. Iatridis, M. Alini, S. Grad, Z. Li

**Affiliations:** 1 AO Research Institute Davos, Davos, Switzerland; 2 Icahn School of Medicine at Mount Sinai, New York, USA; 3 Tokai University School of Medicine, Isehara, Japan; 4 Inselspital, University of Bern, Bern, Switzerland; 5 Shenzhen Key Laboratory of Anti-Aging and Regenerative Medicine, Department of Medical Cell Biology and Genetics, Health Sciences Centre, Shenzhen University, Shenzhen, China; 6 Orthopaedic Institute, Medical College, Soochow University, Suzhou, Jiangsu, China; 7 Collaborative Research Program Annulus Fibrosus Repair, AO Foundation, Davos, Switzerland

**Keywords:** Annulus fibrosus, annular rupture repair, tissue engineering, transforming growth factor β1, polyurethane scaffold, collagen type I hydrogel

## Abstract

Appropriate cell sources, bioactive factors and biomaterials for generation of functional and integrated annulus fibrosus (AF) tissue analogues are still an unmet need. In the present study, the AF cell markers, collagen type I, cluster of differentiation 146 (CD146), mohawk (MKX) and smooth muscle protein 22α (SM22α) were found to be suitable indicators of functional AF cell induction. *In vitro* 2D culture of human AF cells showed that transforming growth factor β1 (TGF-β1) upregulated the expression of the functional AF markers and increased cell contractility, indicating that TGF-β1-pre-treated AF cells were an appropriate cell source for AF tissue regeneration. Furthermore, a tissue engineered construct, composed of polyurethane (PU) scaffold with a TGF-β1-supplemented collagen type I hydrogel and human AF cells, was evaluated with *in vitro* 3D culture and *ex vivo* preclinical bioreactor-loaded organ culture models. The collagen type I hydrogel helped maintaining the AF functional phenotype. TGF-β1 supplement within the collagen I hydrogel further promoted cell proliferation and matrix production of AF cells within *in vitro* 3D culture. In the *ex vivo* IVD organ culture model with physiologically relevant mechanical loading, TGF-β1 supplement in the transplanted constructs induced the functional AF cell phenotype and enhanced collagen matrix synthesis. In conclusion, TGF-β1-containing collagen-PU constructs could induce the functional cell phenotype of human AF cells *in vitro* and *in situ*. This combined cellular, biomaterial and bioactive agent therapy has a great potential for AF tissue regeneration and rupture repair.

## Editor’s note

The Scientific Editor responsible for this paper was Brian Johnstone.

## Introduction

LBP has become one of most common chronic health problems. Approximately 80 % of the population has been affected by LBP at least once in their life, representing a high economic and social burden (Dionne *et al.*, 2006; Hoy *et al.*, 2012; Hoy *et al.*, 2010). IVD degeneration and disc herniation are well-known as leading causes of specific LBP (Roberts *et al.*, 2006). In IVD herniation, the NP protrudes out of the defective AF and irritates and compresses the spinal nerves, resulting in pain and dysfunction. Furthermore, the integrity of the IVD is destroyed, which leads to mechanical property loss (consequently, reduced disc height), inflammation and further disc degeneration (Goupille *et al.*, 1998; Humphreys and Eck, 1999). Microdiscectomy/sequestrectomy is the standard surgical treatment for patients with severe dysfunction or without satisfactory outcomes after conservative therapy. The acute symptoms are relieved by removing herniated tissue. However, AF defects remain unrepaired, which leads to a 10–30 % re-herniation rate resulting in recurrent pain and continuing disc degeneration (Ambrossi *et al.*, 2009; Carragee *et al.*, 2003; Smith *et al.*, 2010). Aggressive discectomy with removal of all NP material reduces the risk of re-herniation but will lead to a post-discectomy syndrome with rapid disc degeneration and pain in nearly one third of the patients (Watters and McGirt, 2009).

Several attempts have been made at AF repair after discectomy, such as direct suture and conglutination with glue; however, outcomes have been unsatisfactory (Ahlgren *et al.*, 2000; Bailey *et al.*, 2013; Guterl *et al.*, 2014; Heuer *et al.*, 2008). The self-healing capacity of the AF tissue is confined due to its avascular nature. Additionally, with the IVD as a load-bearing tissue, the repair of the AF should not only restore its mechanical properties but also reproduce its biological integrity (Chu *et al.*, 2018). Tissue engineering has been demonstrated to be a prospective approach for AF repair in the last few decades (Agnol *et al.*, 2018; Borem *et al.*, 2017; Frauchiger *et al.*, 2018; Hussain *et al.*, 2018; Pirvu *et al.*, 2015). However, appropriate cell sources, growth factors and biomaterials for generation of functional and integrated AF tissue analogues are still unmet needs.

MSCs have been proposed as a suitable cell source for IVD regenerative therapies (Sakai and Grad, 2015). In an ovine lumbar disc degeneration model, injection of bone-marrow-derived MSCs into previously incised AF resulted in a significant improvement in histological, biochemical and MRI analyses (Freeman *et al.*, 2016). Nevertheless, the fate of the implanted cells and their potential to differentiate into AF-like cells remain uncertain. In a previous study, MSCs were transplanted into AF defects of bovine caudal IVDs and cultured in an organ culture model under dynamic load for 14 d, to investigate the phenotype of MSCs after transplantation and their paracrine effect on native disc cells (Pirvu *et al.*, 2015). MSCs showed the ability to positively affect the phenotype of the host disc cells, as indicated by the up-regulation of anabolic genes and down-regulation of catabolic genes. However, no effect was detected on new matrix formation at the AF defect site. This finding demonstrated that non-primed MSCs have limited capacity to differentiate into AF cells *in situ*. An effective cell population with repair ability is required to reconstruct an AF tissue with normal functions. AF cells possess the innate capacity to rebuild the biological properties of tissue-engineered AF analogues (Iu *et al.*, 2017; Yang *et al.*, 2009). However, phenotypic markers of functional AF cells as a guideline for seed cell selection are still missing, except for collagen type I, which is the prominent ECM component in the AF. Recently, Nakai *et al.* (2016) have demonstrated that CD146^+^ murine AF cells deposit more collagen type I-rich ECM as compared with CD146^−^ cells, indicating that CD146 may be a marker of functional AF cells. The present study unravelled further molecular markers of healthy AF cells as compared with NP cells. Then, these markers were used as indicators of functional AF cell induction.

Several studies have shown that TGF-β1 enhances ECM production and cell proliferation of human AF cells in both 2D and 3D *in vitro* cultures (Chou *et al.*, 2016; Gruber *et al.*, 2004). TGF-β1 increases myofibroblast contractility in wound healing, which plays a pivotal role in physiological tissue remodelling (Tomasek *et al.*, 2002). TGF-β1 can also prevent degenerative processes and inhibit inflammatory responses in the dorsal root ganglion and prevent pain development in a rat IVD degeneration model (Zhang *et al.*, 2017). Therefore, the effect of TGF-β1 on the expression of the functional markers in human AF cells was investigated. In addition to 2D and 3D *in vitro* culture systems, TGF-β1-treated AF cells were tested in a preclinical IVD organ culture model to reveal their repair effect *in situ*.

In addition to cell source and growth factor, a biomaterial with appropriate biological and biomechanical compatibility is crucial for successful regeneration of AF tissue *in situ*. PU scaffolds have been reported to have excellent biocompatibility, mechanical property and stability for AF tissue engineering (Agnol *et al.*, 2018; Yeganegi *et al.*, 2010). Whatley *et al.* (2011) developed a PU scaffold to mimic the native shape and structure of the IVD that exhibited elastic behaviour during compressive and shear testing and supported cell growth. Lee *et al.* (2005) and Li *et al.* (2009) have performed research on PU scaffolds with an interconnected pore structure for cartilage tissue engineering. Results showed that PU scaffolds have sufficient elasticity, resiliency and stiffness to endure *in vitro* mechanical loading. Hydrogels such as collagen, agarose, fibrin and alginate are widely used to encapsulate and deliver cells into scaffolds, preventing cell loss from scaffold and enhancing the retention of matrix molecules (Alini *et al.*, 2003). Collagen type I, the main component of AF ECM, has been coated on to plastic, promoting AF cell proliferation and matrix production *in vitro* (Xiao *et al.*, 2017). A crosslinked collagen I hydrogel was injected to repair AF rupture in a punctured rat-tail model. Results showed that collagen gel can preserve the integrity of the AF and NP after needle puncture, while delaying the degeneration of the IVD (Wang *et al.*, 2017). Bowles *et al*. (2010) found that a collagen I hydrogel seeded with ovine AF cells display a capacity for self-assembly of aligned tissue-engineered AF through collagen gel contraction. Those studies indicated a good potential of collagen I hydrogel for AF cells proliferation and AF matrix synthesis. However, the specific phenotypes of the AF cells within these various biomaterials have not yet been reported.

The current study aimed to define the functional markers of healthy AF cells and assess the *in vitro* and *ex vivo* effect of a bioactive agent-biomaterial approach for functional AF cells priming and AF rupture repair. First, the markers of functional AF cells were defined; then, potential cell sources (AF cells) with growth factor (TGF-β1) for functional phenotype induction were assessed; finally, those were combined with biomaterials. PU scaffolds with/without collagen I hydrogel were assessed for their capacity to support and maintain the functional phenotype of AF cells in an *in vitro* 3D culture model and an *ex vivo* preclinical organ culture model. Cell proliferation, matrix production and gene expression were evaluated *in vitro*. *Ex vivo* tests were performed on bovine caudal IVDs with an organ culture system including a mechanical loading bioreactor. The morphology of regenerated tissue and the phenotype of implanted and native disc cells were analysed to assess the repair effect of constructs in an AF defect *in situ*.

## Materials and Methods

### Fabrication of PU scaffold and PU membrane

PU was synthesised using a one-step solution polycondensation as described previously (Gorna and Gogolewski, 2006). Briefly, the monomers and macromers used for the synthesis were HMDI (Sigma-Aldrich), hydroxyl-terminated PCL (Sigma-Aldrich), with an average molecular weight of 530 g/mol and a functionality of 2, and ISO (Sigma-Aldrich). The reactants underwent a one-step polycondensation in solution in the presence of a catalyst. The HMDI : PCL : ISO molar ratio used was 1 : 0.32 : 0.64. The PU obtained had intrinsic viscosity, weight average molecular weight and polydispersity of 1.3 dL/g, 500,000 g/mol and 2.00, respectively.

Membranes were produced by casting the N,N-dimethylformamide solution of the synthesised PU into a flat-bottom glass Petri dish and allowing the solvent to evaporate under a chemical hood. The measured ultimate strength of the PU membrane was 53.0 ± 2.0 MPa, the yield strength 4.9 ± 1.4 MPa and the elongation at break 593.8 ± 57.7 %. The surface of the PU membrane did not present large porosity and was mostly smooth on the top and bottom (Pirvu *et al.*, 2015).

Porous PU sponges with interconnected pore size diameter of 150–300 μm were produced using a salt leaching-phase inverse fabrication extensively reported elsewhere (Boissard *et al.*, 2009; Lee *et al.*, 2005). Water-jet cutting was used to produce cylindrical scaffolds of 3 mm diameter, 4 mm thickness and scaffolds were sterilised by cold ethylene oxide and degassed for 5 d before use. The unconfined compressive stiffness of the prepared scaffolds was approximately 20 kPa (Grad *et al.*, 2003).

### Isolation and expansion of human AF cells

Human IVD tissue was harvested with ethical approval (Cantonal Ethics Commission Bern 2016) and written patient consent from traumatic injured IVDs (30–55 years old, 6 male donors), which were classified as mildly degenerated (Pfirrmann grade 2–3). To ensure purity of AF samples, AF tissue was collected after removing all the adjacent NP tissue and cartilage EP. The collected AF tissue was incubated with red blood cell lysis buffer [155 mM NH_4_Cl, 10 M KHCO_3_ and 0.1 mM EDTA in Milli-Q water] for 5 min on a shaker at room temperature and, then, washed with PBS. Then, the chopped tissue was enzymatically digested for 1 h with 0.2 % w/v Pronase (Roche) in αMEM (Gibco), followed by 12–14 h at 37 °C in 130 U/mL collagenase type II (Worthington) in αMEM/10 % FBS (PAN Biotech, Aidenbach, Germany). Single-cell suspension was obtained by filtering through a 100 μm cell strainer. Next, cells were seeded at a concentration of 10,000 cells/cm^2^ and expanded with αMEM supplemented with 10 % FCS, 100 U/mL penicillin and 100 mg/mL streptomycin (1 % P/S, Gibco). Incubation conditions were set to 5 % CO_2_ at 37 °C at a hypoxic condition of 2 % O_2_. Culture medium was changed twice a week and cells were detached at ~ 80 % confluence using a dissociation buffer composed of 0.05 % trypsin/EDTA (Gibco) and 0.01 % collagenase P (Roche) for 5 min at 37 °C. Cells were sub-cultured at a cell density of 3,000 cells/cm^2^ for expansion in the same medium as above. Passage 2 AF cells were used in the present study.

### AF cells, 2D and 3D culture *in vitro*

#### 2D culture in vitro

Passage 2 human AF cells were treated with either basal medium [αMEM, 5 % FBS, 1 % ITS+ (BD Biosciences), 1 % P/S] or TGFβ medium [basal medium with 5 ng/mL recombinant human TGF-β1 (Fitzgerald, Acton, MA, USA)] for 4 d. Then, cells were dissociated for gene expression analysis, contractility study or flow cytometry analysis. AF cells pre-treated with TGFβ medium for 4 d were used for *in vitro* 3D culture experiments and *ex vivo* organ culture experiments.

#### 3D culture in vitro

PU scaffolds were pre-wetted for 1 h in αMEM with 10 % FBS under vacuum conditions. Medium was completely aspirated from the scaffolds, which were placed into 0.5 mL protein-low-binding Eppendorf tubes. Tubes were pre-coated for 1 h at 37 °C with 1 % BSA (Gibco). TGF-β1-treated AF cells were harvested and resuspended with medium or Corning® Collagen I, rat tail solution at a cell density of 2 × 10^5^ cells per 30 μL. The final concentration of the collagen I hydrogel was 1.81 mg/mL. For the TGF-β1 containing group, 5 ng TGF-β1 was added within the AF-cells-collagen I solution suspension. Cell suspension in medium or collagen I solution was dropped onto the scaffold (30 μL per scaffold). Scaffolds were compressed mildly with forceps to allow cell suspension infiltration into the scaffold, then incubated for 1 h at 37 °C to allow cell adhesion and collagen-hydrogel gelation. Next, constructs were transferred into a 24-well plate and cultured at 37 °C, 5 % CO_2’_ 2 % O_2_ in high-glucose DMEM supplemented with 1 % P/S, 2 % FBS, 50 mg/mL L-ascorbic acid 2-phosphate (Sigma-Aldrich), 1 % ITS+ and 1 % NEAA (Gibco). The medium volume was 1 mL per scaffold and it was replaced twice a week. After 7 d of culture, scaffolds were collected for gene expression analysis, DNA and GAG quantification and toluidine blue staining. For the *ex vivo* organ culture study, constructs were immediately implanted into the AF defect after gelation of the collagen type I hydrogel.

### Bovine caudal IVD dissection

Caudal IVDs were harvested from 6–12-month-old calves obtained from a local abattoir after sacrifice. Disc dissection was performed as described previously (Lang *et al.*, 2018). IVDs with cartilage EPs were isolated using a band saw, then scraped using a scalpel to remove the vertebral bone and growth plate and ensure two parallel planes of disc. EP surfaces were cleaned with Ringer’s balanced salt solution using a Pulsavac Wound Debridement Irrigation System (Zimmer, Minneapolis, MN, USA) to remove cutting debris and blood clots. IVDs were pre-washed in PBS with 10 % P/S (Gibco) at room temperature for 20 min, then cultured at 37 °C 5 % CO_2_ in 6-well plates with 7.5 mL IVD culture medium: high-glucose DMEM supplemented with 1 % P/S, 50 mg/mL Primocin (Invitrogen), 2 % FBS, 50 mg/mL ascorbate-2phosphate, 1 % ITS+ and 1 % NEAA. Culture medium was replaced every day.

Different parts of healthy IVD tissue, including NP tissue (gel-like inner core of 6–8 mm diameter), outer AF tissue (oAF, distinguishable lamellar AF tissue, ring at thickness ~ 4 mm) and inner AF tissue (iAF, located between NP and oAF) from 6 bovine tails (age 6–12 months) were collected for gene expression analysis on day 0, to measure the expression level of potential AF markers (Table 1).

### Annulotomy model for preclinical testing of AF repair therapy in whole-organ IVD culture

24 caudal IVDs were dissected from 4 bovine tails (age 6–12 months). Day 0 control group comprised 4 IVDs. AF defect repair groups comprised 6 IVDs per group and consisted of 1) an acellular PU scaffold-collagen hydrogel (PU-Col), 2) PU-Col with TGF-β1 pre-treated AF cells (PU-Col-AFCs) and 3) PU-Col with TGF-β1 pre-treated AF cells and 5 ng TGF-β1 (PU-Col-AFCs-TGFβ). Within the 6 replicates in each group, 4 IVDs were used for gene expression analysis of human AF cells in scaffold and native IVD tissue and 2 IVDs were used for safranin O/fast green staining. 2 IVDs served as non-repair negative control on day 15 and were used for safranin O/fast green staining.

The IVD AF defect was created with a biopsy punch (diameter 3 mm, length 7 mm). Full thickness of AF tissue and some NP tissue were removed. Then, the defect was refilled with Different scaffolds, as described above. A PU membrane (12 × 7 mm) was affixed to the IVD with 2 μL of EPIGLU® (Meyer-Haake GmbH, Ober-Mörlen, Germany) surrounding the defect area, to maintain the scaffold within the defect. IVDs were cultured in IVD culture medium and loaded with physiological compressive loading for 1 h at 0.02–0.2 MPa, 0.2 Hz daily within a bioreactor (Lang *et al.*, 2018). The disc height was measured using a caliper at 2 positions to calculate percentage change normalised to day 0 after dissection. After 14 d of culture, IVDs were collected for evaluations.

### Flow cytometry analysis

TGF-β1-treated and -non-treated AF cells were harvested by trypsinisation. Single-cell suspension was prepared at a cell density of 1 × 10^5^ cells per 100 μL staining buffer (PBS with 0.2 % BSA and 1 mM EDTA). Cell suspensions were incubated at 4 °C in the dark for 30 min with 10 μL fluorescence-conjugated mouse monoclonal anti-human CD146 antibody (CD146-APC, human, Miltenyi Biotec) or 10 μL mouse IgG1-APC (isotype control, Miltenyi Biotech). After incubation and washing, DAPI at a final concentration of 0.1 μg/mL was added for live/dead staining. Flow cytometric analysis was performed on a FACS AriaIII (BD Biosciences) and at least 30,000 events per sample were recorded. Data analysis was performed using BD FACSDiva software. A gating strategy was used to exclude dead cells and cell doublets.

### Gene expression analysis

RNA isolation from 2D-cultured AF cell samples was performed using TRI reagent (Molecular Research Centre Inc., Cincinnati, OH, USA) according to the manufacturer’s protocol. AF cells in PU scaffold-collagen type I hydrogel constructs were placed into 1 mL TRI reagent with 5 μL polyacryl carrier (Molecular Research Centre Inc.) and homogenised by a tissue-lyser (Retsch GmbH, Haan, Germany). After centrifugation at 12,000 ×*g* for 15 min, the supernatant was collected in a fresh EP tube and the RNA isolation was performed according to the manufacturer’s protocol. Native IVD tissues, including NP and AF in intact discs, as well as AF tissue adjacent and opposite to the repair constructs, were collected on day 0 and 14. Tissues of 150–200 mg/sample were cut into small pieces and snap-frozen by liquid nitrogen and hammering (Caprez Stephanie, 2018). Then, tissues were transferred into 3 mL TRI reagent with 15 μL polyacryl carrier. Samples were homogenised using a tissue-lyser. After centrifugation, the supernatant was extracted by phase separation by adding 100 μL bromochloropropane per 1 mL of TRI reagent. The aqueous phase after centrifugation was transferred to a fresh tube and mixed with the same volume of 70 % ethanol. Then, RNA isolation was performed using the QIAGEN RNeasy MINI kit according to the manufacturer’s protocol. Reverse transcription was performed using SuperScript® VILO™ cDNA Synthesis Kit (Invitrogen).

qRT-PCR was conducted on QuantStudio6 System (Applied Biosystems). Sequences of the primers and probes used in qRT-PCR of bovine and human cells/tissue are listed in Table 1. *RPLP0* ribosomal RNA was used as endogenous control. Data were analysed using the 2^−ΔΔCT^ method.

### Cell contraction functionality assay

Contractility of TGF-β1-treated and -non-treated cells was evaluated by a cell contraction assay in collagen I hydrogel. A 24-well plate was coated with 1 % BSA and incubated at 37 °C for at least 1 h. Cells from 3 donors were encapsulated in 1.81 mg/mL type I collagen with 4 technical replicates at a seeding density of 1.5 × 10^5^ cells/mL. Cells were cultured at 37 °C 2 % O_2_ in αMEM with 10 % FBS. After 24 h, gels were photographed (Canon PowerShot SX50 HS) and well and gel diameters measured using NIS-Elements D 3.2 (NIKON Japan). Two gel diameters were averaged to calculate the gel area. The gel area after 24 h was divided by the well area, calculated from the well diameter.

### Toluidine blue staining

Cell distribution and ECM deposition within PU scaffold with or without hydrogel were evaluated by toluidine blue staining. Samples at day 1 and 7 were snap-frozen in cryo-compound (Leica). Vertical sections of scaffold were made at a thickness of 12 μm. Slides were fixed in 70 % methanol for 10 min, 100 % methanol for 10 min, dried overnight, stained with 0.1 % toluidine blue for 2 min and rinsed with deionised water (4–5 times).

### Safranin O/fast green staining

IVD samples after 14 d of culture were snap-frozen in cryo-compound after removal of the cartilage EP from one side. IVD transverse cryo-sections were made at a thickness of 12 μm. Slices were fixed as above, stained with 0.1 % safranin O and 0.02 % fast green to reveal proteoglycan and collagen deposition, respectively, and counterstained with Weigert’s haematoxylin to reveal cells distribution (Lang *et al.*, 2018).

Cell density in the implanted constructs was calculated using the safranin O/fast green-stained images. High-magnification images (676 × 536 μm) were taken at 6 random positions of each slide, with 2 slides per IVD and 2 IVDs per group. In total, cells from 24 images of each group were counted by Image J 1.52a (NIH).

### GAG and DNA content measurement

Cell-scaffold constructs after 7 d of culture were digested overnight at 56 °C with 0.5 mg/mL proteinase K (Roche, Mannheim, Germany) solution. DNA content was measured using the PicoGreen kit (Invitrogen) according to the manufacturer’s instruction. GAG content was determined by dimethylmethylene blue assay (1.9-DMMB; Sigma-Aldrich).

### Statistical analysis

Statistical analyses were performed using GraphPad Prism 7 software (GraphPad Software, Inc., La Jolla, CA, USA). D’Agostino-Pearson omnibus normality test was used to define whether the data were normally distributed. For data that were normally distributed, unpaired *t*-test was used to determine differences between two groups; one-way ANOVA was used to determine differences between three or more groups. For data that were not normally distributed, Mann-Whitney U test was used to determine differences between two groups; Kruskal Wallis test was used to determine differences between three or more groups. *p* < 0.05 was considered statistically significant.

## Results

### Phenotype markers of AF cells

**Fig. 1.**
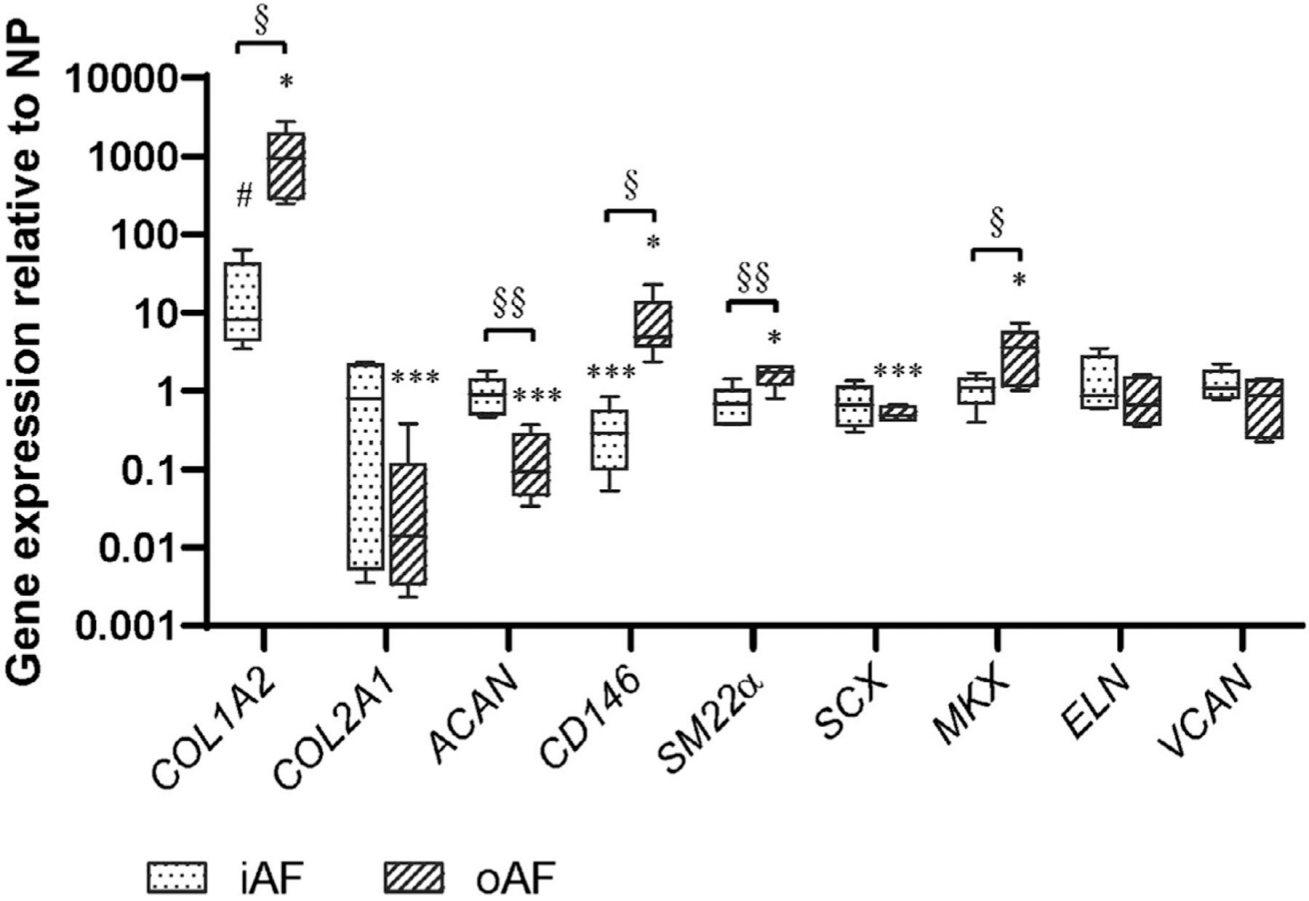
Relative mRNA expression in NP, iAF and oAF from healthy bovine IVDs. Data were normalised to the expression level of NP tissue. *n =* 6, ^#^ *p* < 0.1, * *p* < 0.05, *** *p* < 0.001 *vs*. gene expression level in NP, ^§^ *p* < 0.05, ^§§^ *p* < 0.01 comparing iAF and oAF.

Phenotype markers of AF cells were defined by comparison of the gene expression levels in Different parts of bovine IVD tissue. Compared with NP tissue and iAF, oAF expressed higher levels of *COL1A2* (*p* < 0.05), *CD146* (*p* < 0.05), *SM22α* (*p* < 0.05) and *MKX* (*p* < 0.05) (Fig. 1), which were, therefore, defined as phenotype markers of healthy and functional AF cells. Furthermore, oAF tissue expressed lower levels of *COL2A1* (*p* < 0.001), *ACAN* (*p* < 0.001) and *SCX* (*p* < 0.001) as compared with NP tissue. Interestingly, the transient zone iAF tissue showed a comparable gene expression pattern to NP tissue, except for lower levels of *CD146* (*p* < 0.001).

### Induction of the functional human AF cell phenotype for AF repair – *in vitro* 2D culture

**Fig. 2.**
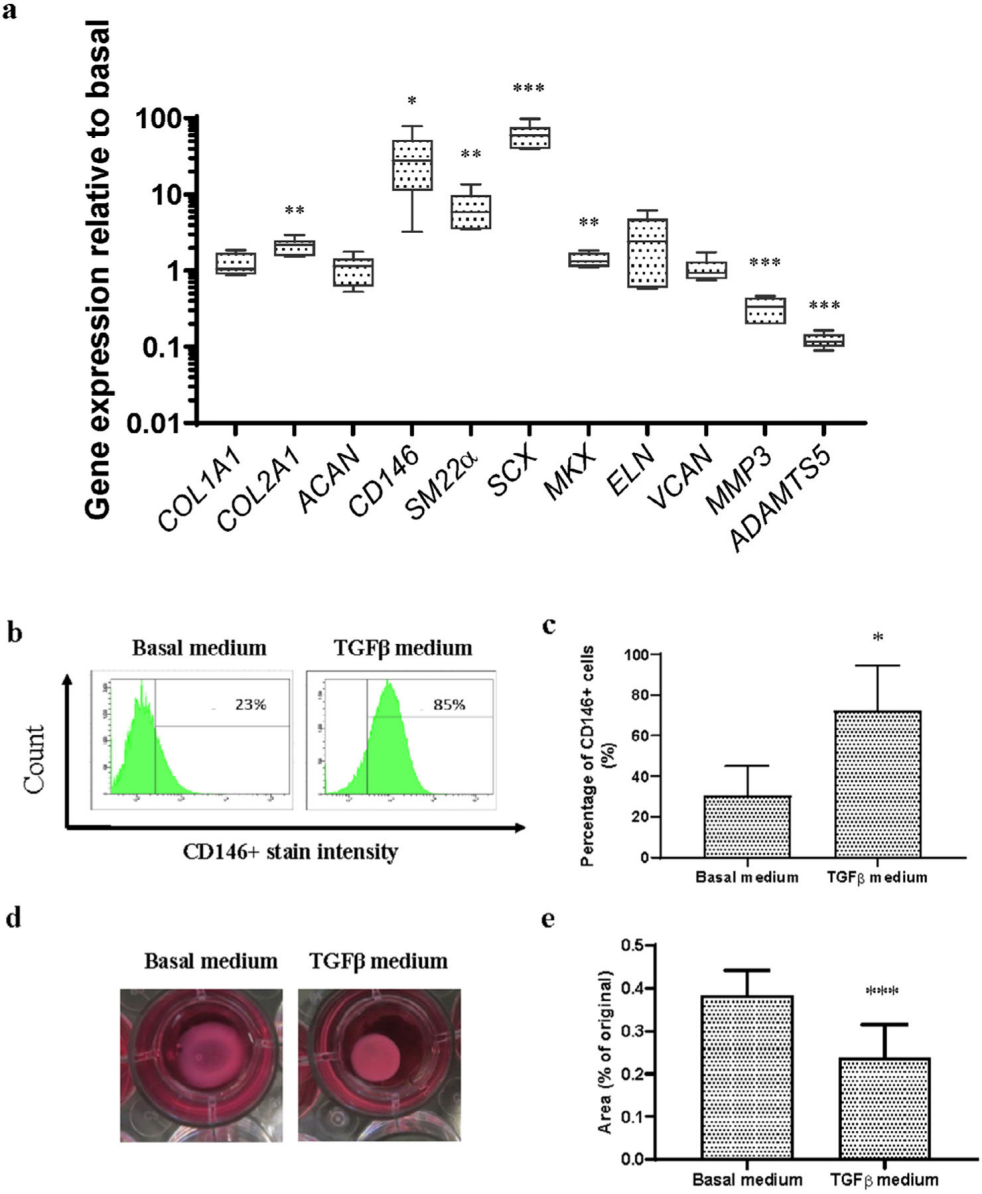
Phenotype of human AF cells after treatment with TGF-β1 for 4 d. (**a**) Relative mRNA expression, data were normalised to basal medium. (**b**) Representative and (**c**) average percentage of CD146^+^ cells detected by flow cytometry. (**d**,**e**) Cell contractibility of AF cells cultured with basal or TGFβ medium. Mean + SD; *n =* 4, * *p* < 0.05, ** *p* < 0.01, *** *p* < 0.001 *vs*. cells treated with basal medium.

To evaluate the functional phenotype of human AF cells induced by TGF-β1, gene expression levels of the AF markers were measured in human AF cells cultured with or without 5 ng/mL TGF-β1 for 4 d, since cells reached 70–80 % confluency after 4 d of culture, which is the optimal time point for cell trypsinisation and seeding on to the scaffold (Fig. 2a). mRNA levels of *CD146* (*p* < 0.05), *SM22a* (*p* < 0.01), *SCX* (*p* < 0.01), *MKX* (*p* < 0.01) and *COL2A1* (*p* < 0.01) were significantly upregulated by TGF-β1 treatment. CD146 upregulation at the protein level was confirmed by flow cytometry (Fig. 2b,c). The percentage of CD146^+^ cells in 4 donors was 30.50 ± 14.55 % in basal medium, which increased to 72.25 ± 22.02 % in TGFβ medium. In contrast, ECM degrading enzymes, *MMP3* (*p* < 0.001) and *ADAMTS5* (*p* < 0.001), were downregulated by TGF-β1 treatment (Fig. 2a). Cell contractility was determined in collagen I hydrogel by measuring the surface area of the collagen gel after 24 h of incubation, as a functionality test. TGFβ medium induced a larger reduction of the gel area (22.8 ± 8.1 %) as compared with basal medium (38.3 ± 5.9 %) (Fig. 2d,e), which indicated a higher cell contractility after TGF-β1 treatment.

### Preservation of the functional human AF cells phenotype for AF repair – *in vitro* 3D culture

To assess cell phenotype and tissue construction capabilities of induced functional human AF cells in an *in vitro* 3D model, human AF cells pre-treated with TGF-β1 for 4 d were seeded on to PU scaffold only (PU-AFCs) or PU scaffold-collagen I hydrogel constructs with (PU-Col-AFCs-TGFβ) or without (PU-Col-AFCs) 5 ng TGF-β1 supplement. Gene expression was measured at day 7, cell proliferation and matrix production both at day 1 and 7 after cell seeding, to investigate the effect of the cell carrier on functional AF cell phenotype maintenance and AF tissue regeneration.

**Fig. 3.**
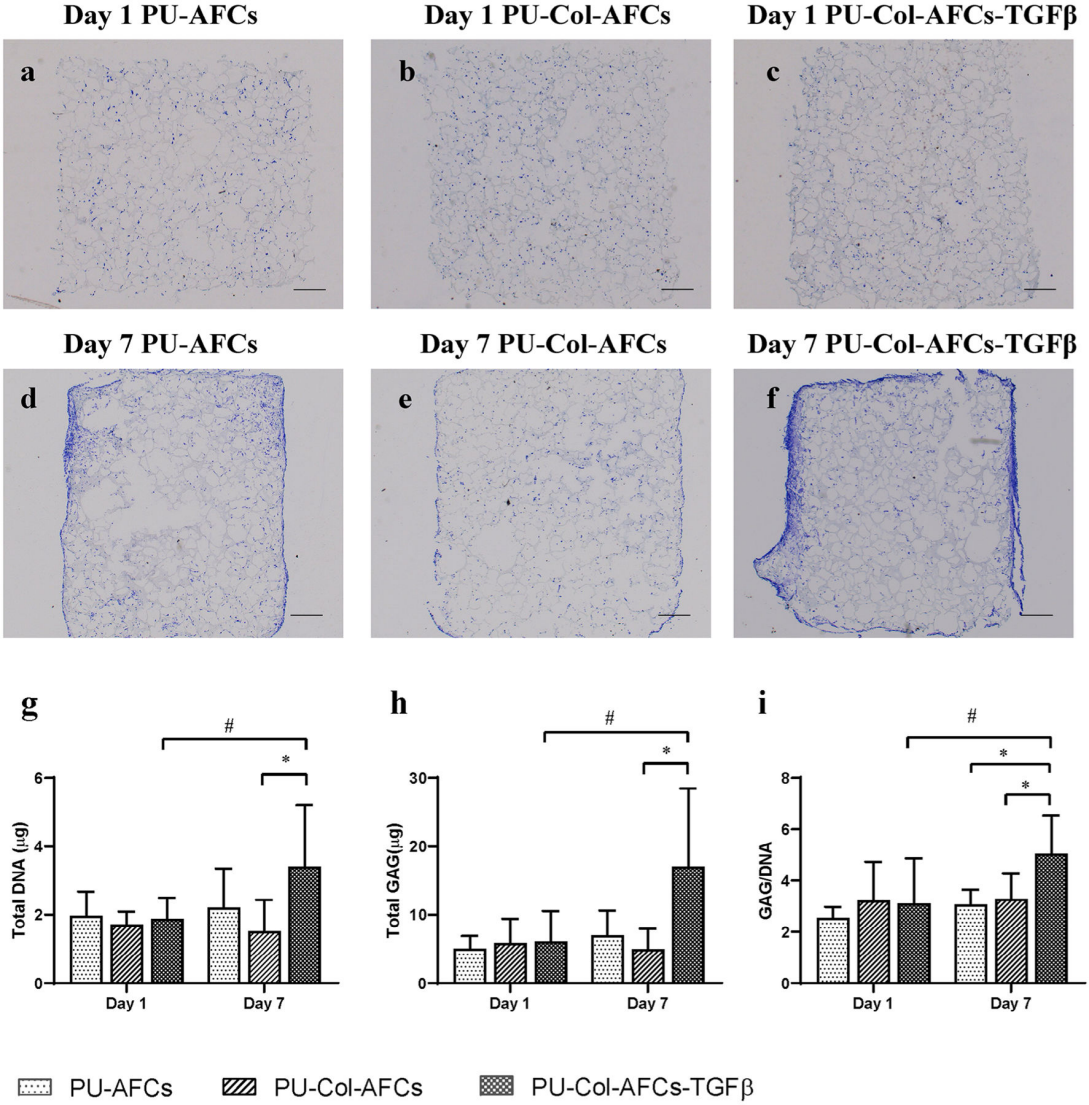
3D culture of human AF cells *in vitro*. (**a**-**f**) Representative toluidine-blue-stained sections of scaffolds. TGF-β1-pre-treated human AF cells seeded on to (**a**,**d**) scaffolds PU-AFCs, (**b**,**e**) PU-Col-AFCs and (**c**,**f**) PU-Col-AFCs-TGFβ after 1 and 7 d. Scale bar: 500 μm. (**g**) DNA content, (**h**) GAG content and (**i**) GAG/DNA ratio in scaffolds after 1 and 7 d of culture. Mean + SD, *n =* 6, ^#^ *p* < 0.1, * *p* < 0.05.

AF cells were evenly distributed in the PU scaffold both with or without collagen I hydrogel at day 1 after seeding (Fig. 3a–c). After 7 d of culture, higher cell density and more intense ECM staining were observed in PU-Col-AFCs-TGFβ (Fig. 3f) as compared to the other two groups (Fig. 3d,e), especially around the outer surface of the PU scaffold. Furthermore, DNA and GAG content and GAG/DNA ratio were significantly higher in the PU-Col-AFCs-TGFβ scaffold when compared with PU-Col-AFCs (Fig. 3g–i). When comparing day 7 with day 1, DNA and GAG content and GAG/DNA ratio increased only in the PU-Col-AFCs-TGFβ group.

**Fig. 4.**
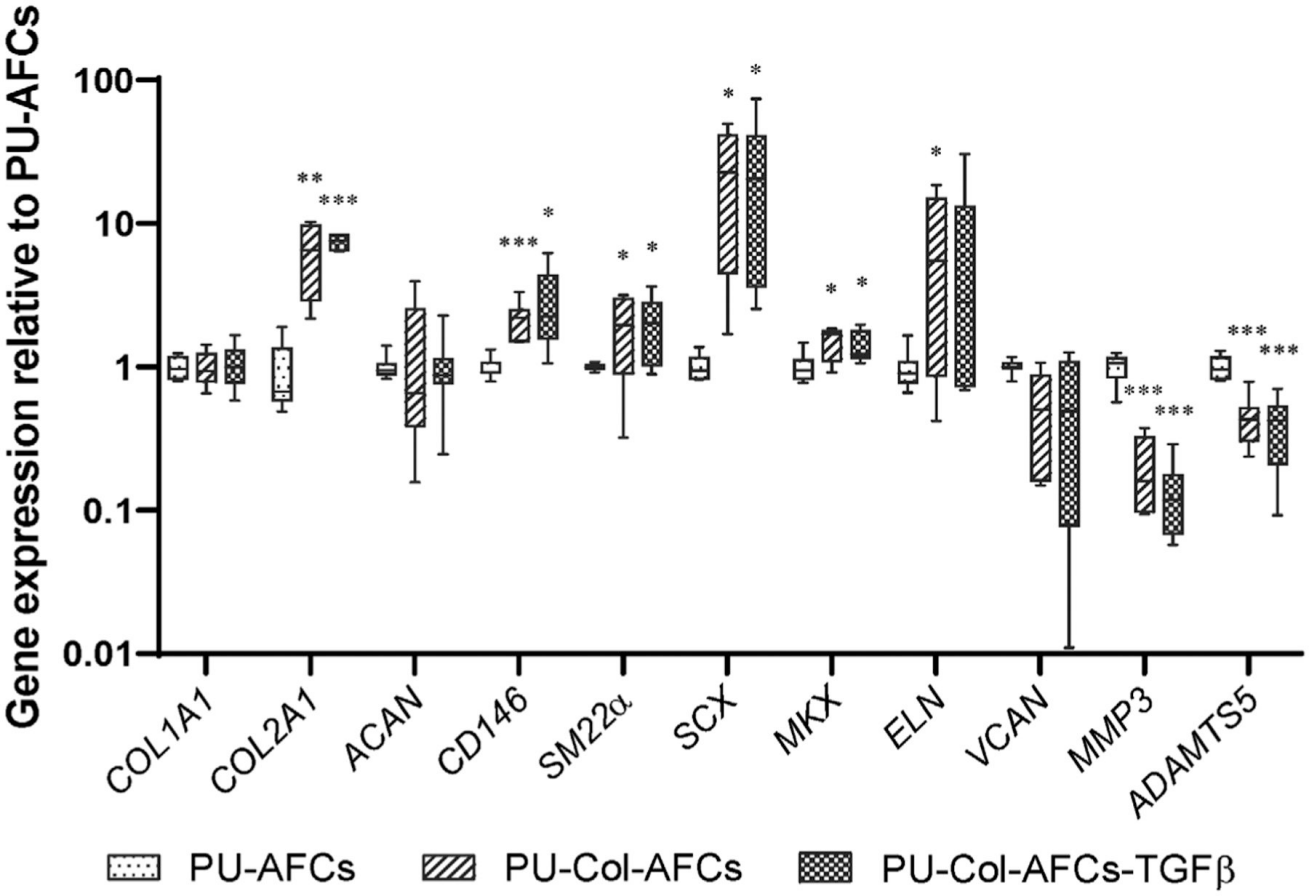
Gene expression of human AF cells 3D-cultured *in vitro*. Relative mRNA expression of TGF-β1-pre-treated human AF cells seeded on to PU scaffold only (PU-AFCs), PU scaffold-collagen I hydrogel construct (PU-Col-AFCs) and PU-Col scaffold supplemented with 5 ng TGF-β1 (PU-Col-AFCs-TGFβ) after 7 d of culture. Data were normalised to expression level of PU-AFCs. *n =* 6, * *p* < 0.05, ** *p* < 0.01, *** *p* < 0.001 *vs*. gene expression levels of PU-AFCs.

Gene expression levels were evaluated in PU-AFCs, PU-Col-AFCs and PU-Col-AFCs-TGFβ after 7 d of culture and data were normalised to PU-AFCs. AF cells in PU-Col-AFCs and PU-Col-AFCs-TGFβ expressed higher levels of *COL2A1*, *CD146*, *SM22a*, *SCX* and *MKX* and lower levels of *MMP3* and *ADAMTS5* when compared with PU-AFCs (Fig. 4). TGF-β1 supplementation into the PU scaffold-collagen I hydrogel did not further affect the gene expression profile of AF cells as compared with the PU-Col-AFCs group.

### AF rupture repair – preclinical testing in *ex vivo* IVD organ culture system

**Fig. 5.**
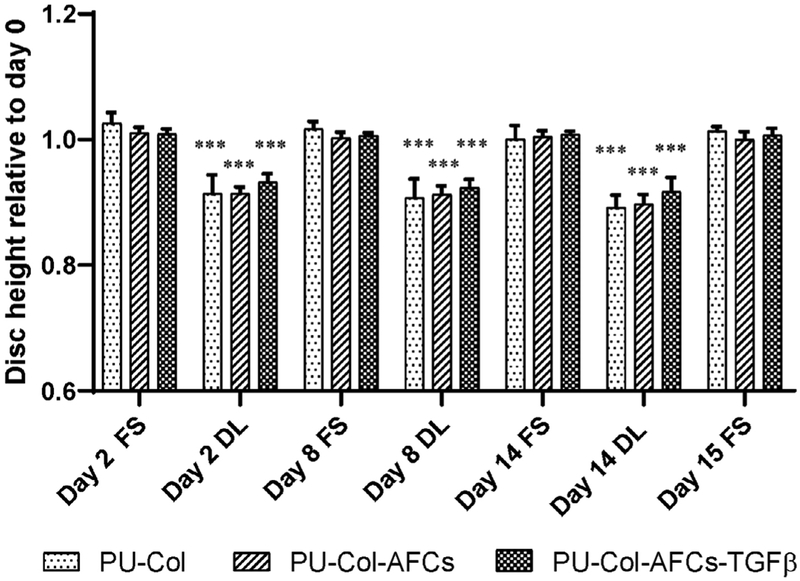
Disc height change during 15 d organ culture. Disc height change relative to initial dimension after dissection (day 0) at Different time points: after free swelling culture overnight and after dynamic loading over 15 d of organ culture. PU-Col: annulotomised IVDs implanted with PU-Col scaffold; PU-Col-AFCs: PU-Col scaffold seeded with AF cells; PU-Col-AFCs-TGFβ: PU-Col-AFCs supplemented with TGF-β1. Mean + SD, *n =* 6. *** *p* < 0.001 *vs*. disc height on day 0.

To test the property of the constructs in a preclinical organ culture model, annulotomised IVDs were repaired with PU scaffold-collagen hydrogel (PU-Col) or PU-Col with AF cells with (PU-Col-AFCs-TGFβ) or without (PU-Col-AFCs) 5 ng TGF-β1. Then, IVDs were cultured under physiological loading condition for 2 weeks, mimicking the *in situ* microenvironment for testing of AF rupture repair. The disc height change was measured during the whole culture period (Fig. 5). At day 2, disc height slightly increased for all groups after overnight free swelling as compared with day 0. Dynamic loading for 1 h caused a physiological disc height loss in all groups repaired with implants: PU-Col 8.6 ± 2.9 %, PU-Col-AFCs 8.6 ± 1.0 %, PU-Col-AFCs-TGFβ 6.8 ± 1.4 %. After 14 d of repetitive dynamic loading, IVDs showed about 2 % of further disc height loss: PU-Col 10.8 ± 1.9 %, PU-Col-AFCs 10.2 ± 1.5 %, PU-Col-AFCs-TGFβ 8.4 ± 2.2 %. However, all IVDs recovered after free swelling. After 15 d culture with daily dynamic loading, no significant disc height loss was observed. Cell implantation and TGF-β1 supplementation did not show any effect on disc height change under dynamic loading.

**Fig. 6.**
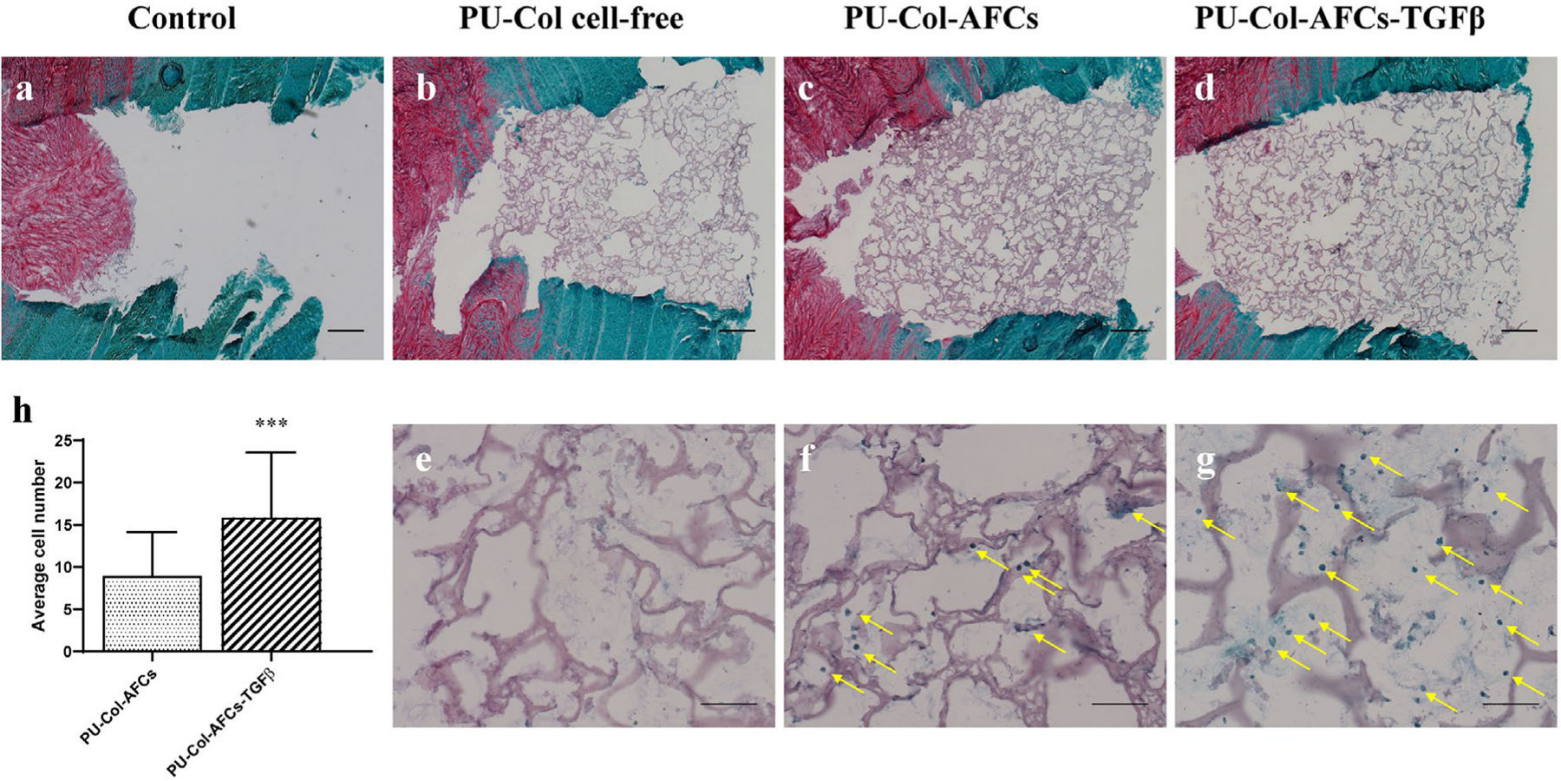
Representative safranin O/fast green-stained sections of IVDs after 15 d organ culture. (**a**) Annulotomised discs and annulotomised discs repaired with (**b**,**e**) PU-Col cell-free, (**c**,**f**) PU-Col scaffold seeded with TGF-β1-pre-treated AF cells (PU-Col-AFCs) and (**d**,**g**) PU-Col-AFCs supplemented with TGF-β1 (PU-Col-AFCs TGFβ), cultured for 15 d under dynamic load. (**h**) Cell numbers counted from the high magnification images in safranin O/fast green-stained sections. Mean + SD, *n =* 24, *** *p* < 0.001 *vs*. PU-Col-AFCs. Scale bar: (**a**-**d**) 500 μm, (**e**-**g**) 100 μm. Yellow arrows indicate cells.

Safranin O/fast green staining of transverse IVD sections after 15 d of culture is shown in Fig. 6. Part of the NP tissue protruded into the AF defect region in the injury control group without repair (Fig. 6a). In all IVDs repaired with PU scaffold-collagen hydrogel (PU-Col cell-free, PU-Col-AFCs and PU-Col-AFCs-TGFβ), NP tissue was maintained in its native position (Fig. 6b–d). When the AF defect was repaired with PU-Col scaffold without AF cells (Fig. 6b,e), after 15 d of culture, no fast-green staining could be detected at the defect site. When the defect was repaired with PU-Col scaffold with AF cells (Fig. 6c,f), AF cells were found in the scaffold after 15 d of culture. Collagen staining with fast green was also detected at the defect site. Furthermore, when TGF-β1 was supplemented in the collagen hydrogel (Fig. 6d,g), a higher cell density and stronger collagen staining intensity was observed at the defect site. The average cell number in high magnification visual fields was significantly higher in PU-Col-AFCs-TGFβ as compared with PU-Col-AFCs (*p* < 0.001) (Fig. 6h).

**Fig. 7.**
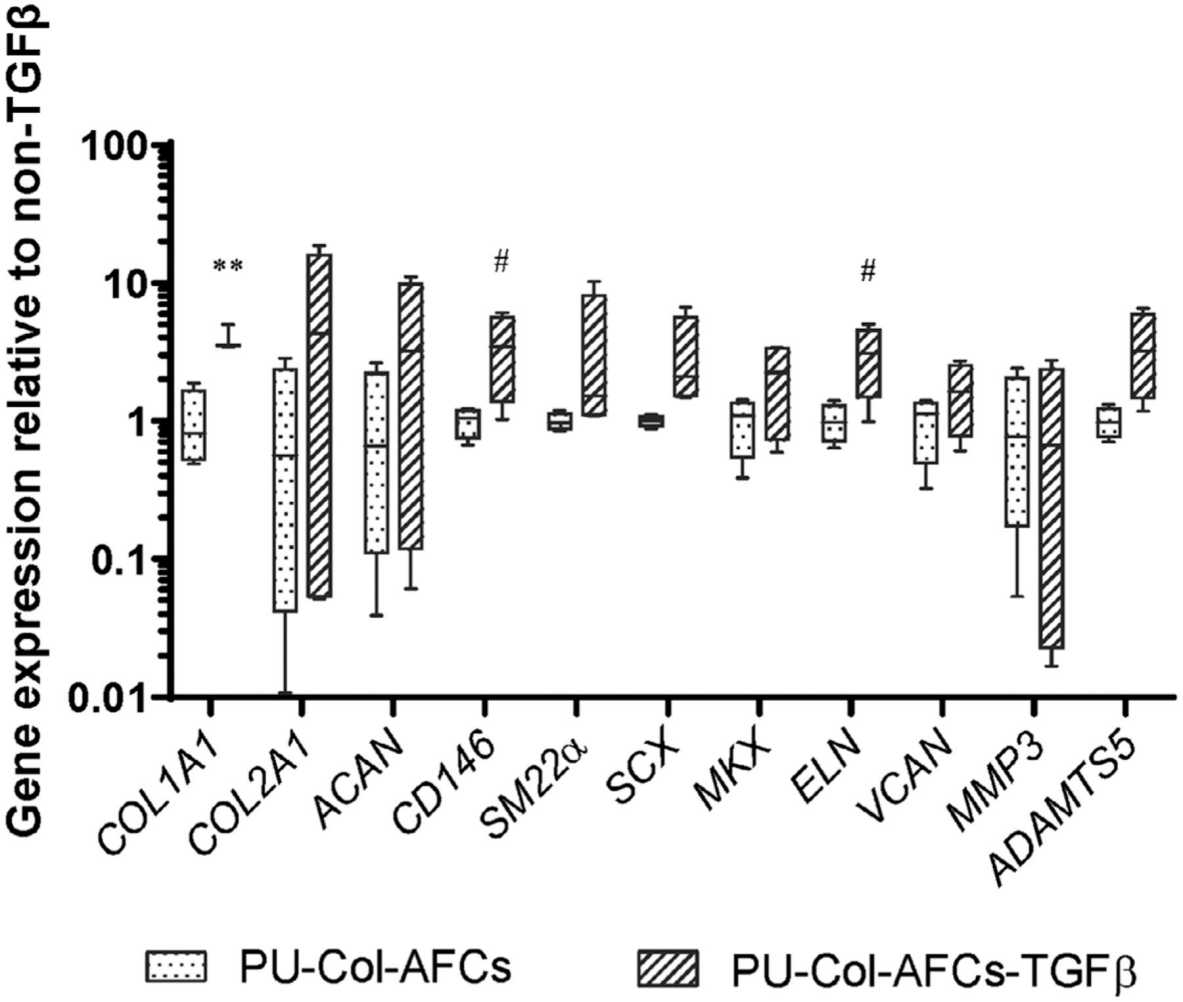
Relative mRNA expression of human AF cells which were encapsulated in implants after 15 d organ culture. AF cells were encapsulated in PU-Col scaffold (PU-Col-AFCs) or PU-Col scaffold supplemented with TGF-β1 (PU-Col-AFCs-TGFβ) and implanted into AF defect, cultured for 15 d *in situ* with dynamic loading. Data were normalised to expression level of PU-Col-AFCs. *n =* 4, ^#^ *p* < 0.1, ** *p* < 0.01 *vs*. gene expression levels of PU-Col-AFCs.

**Fig. 8.**
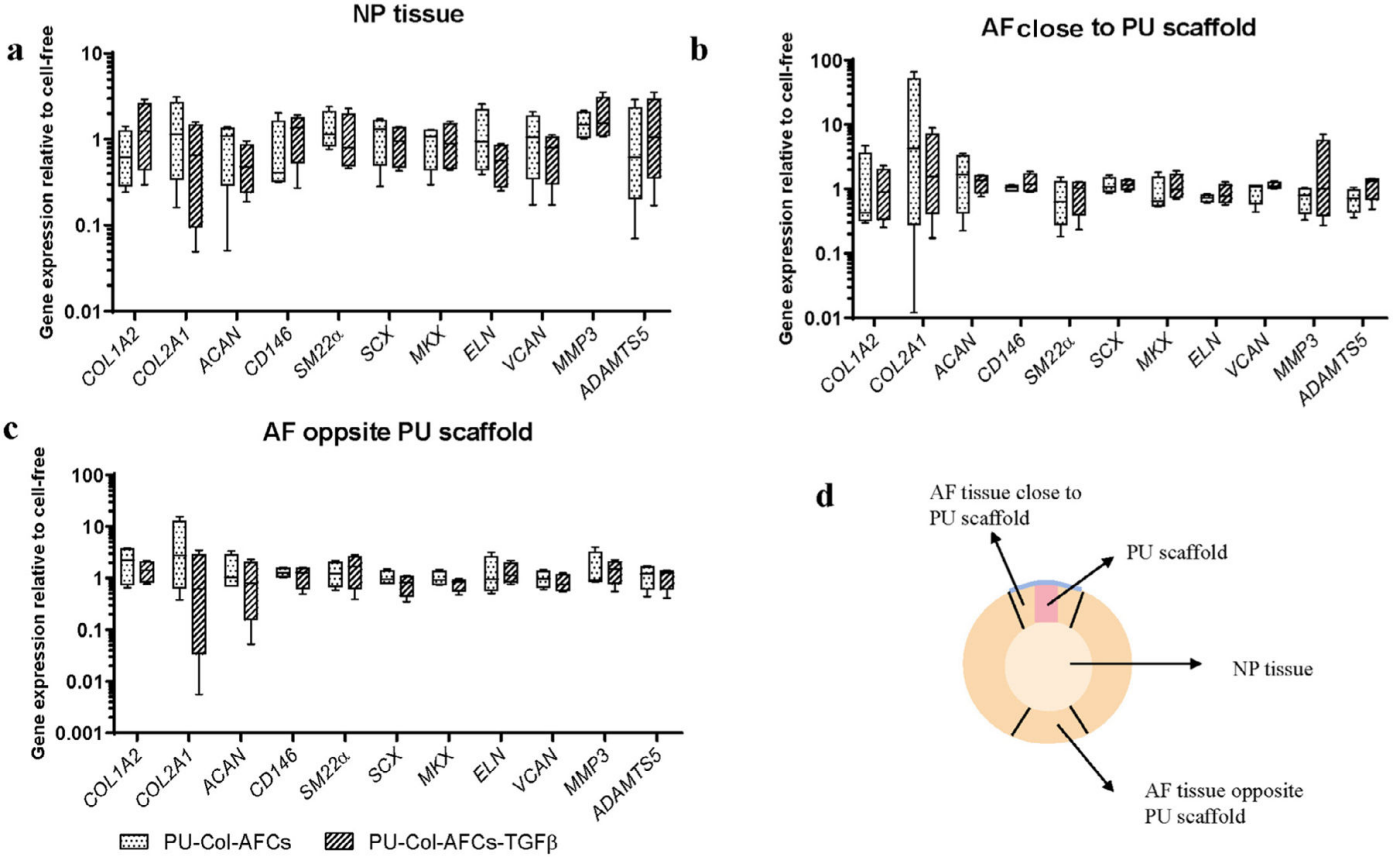
Relative mRNA expression of host disc tissue after 15 d of organ culture. Relative mRNA expression in (**a**) NP, (**b**) adjacent AF and (**c**) distal AF tissue of discs repaired with PU-Col cell-free (PU-Col), PU-Col scaffold seeded with TGF-β1-pre-treated AF cells (PU-Col-AFCs) and PU-Col-AFCs supplemented with TGF-β1 (PU-Col-AFCs-TGFβ) after 15 d of organ culture with dynamic loading. Data were normalised to the expression level of PU-Col. *n =* 4. (**d**) Transverse schematic view of discs implanted with scaffolds and disc tissue collected for gene expression analysis.

Gene expression levels of implanted AF cells after 15 d of organ culture was analysed (Fig. 7). Results showed that *COL1A1* (*p* < 0.01) expression was significantly higher in PU-Col-AFCs-TGFβ as compared with PU-Col-AFCs, which correlated with the fast green staining results. There was also a trend for increased *CD146* (*p* < 0.1) and *ELN* (*p* < 0.1) expression by TGF-β1 supplementation in the collagen gel. Gene expression levels in the native IVD tissues, including AF close to PU scaffold, NP tissue and AF opposite PU scaffold, were also measured after 15 d of organ culture. No significant difference was found among the 3 repaired groups (Fig. 8).

## Discussion

There is an unmet clinical need for AF rupture repair, especially for large AF defects. Tissue engineering AF for large AF rupture repair is considered to be a promising approach to prevent disc re-herniation and associated discogenic pain and to reconstitute the disc biological and mechanical function (Chu *et al.*, 2018; Iatridis *et al.*, 2013; Sloan *et al.*, 2018).

A clear understanding of markers related to the healthy and functional AF cell phenotype is fundamental to define proper cell sources for AF tissue regeneration. However, there is surprisingly limited research on AF cell phenotype so far (Pattappa *et al.*, 2012; Torre *et al.*, 2019). In the current study, the expression profile of several potential phenotypic markers of functional AF cells was measured in healthy oAF tissue as compared with iAF and NP tissue. *COL1A2*, *CD146*, *SM22α* and *MKX* were most highly expressed in the oAF (Fig. 1). Thus, they were defined as markers of functional AF cells (Bron *et al.*, 2009; Costi *et al.*, 2007; Nakamichi *et al.*, 2016; Schmidt *et al.*, 2007). Each of these markers had previously been shown to be related to AF tissue function. Collagen type I expression at both gene and protein level is a well-known marker of AF cells in all species related with the tissue ECM property, since the proportion of collagen type I increases from the NP towards the outer AF (Bron *et al.*, 2009). MKX expression at both gene and protein level has recently been identified as a transcription factor for AF cell Differentiation, where MKX knock-out mice showed a much smaller collagen fibril diameter and a more rapid IVD degeneration as compared with the wild type mice (Nakamichi *et al.*, 2016). Nakai *et al.* (2016) found that CD146 and SM22α are co-expressed in the mouse oAF tissue, which may be correlated with the maximum shear strain in the oAF tissue under lateral bending of IVD (Costi *et al.*, 2007; Schmidt *et al.*, 2007). Interestingly, the expression level of *CD146* in iAF was significantly lower than in NP. The exact reason is unclear, since few studies have reported CD146 expression in NP tissue and in iAF tissue. Milinos *et al.* (2015) found that 22.3 % of bovine primary NP cells are positive for CD146 FACS staining, while in human primary NP cells, CD146 expression is not detected on the cell surface (Sakai *et al.*, 2012). Based on the results from the current study, CD146 may serve as a candidate marker to distinguish NP and iAF. However, further studies are needed with a larger sample size and the protein expression should be confirmed. The current study, for the first time, systematically measured and identified these molecules as a marker pool for functional AF cells. These markers were defined in healthy bovine tissue due to very limited access to healthy human IVD tissue with good zonal structures. Some of the markers have been reported to be relevant to AF function in human and other species, as discussed above. Therefore, they were expected to be similar between bovine and human species and were used as a guideline for cell induction with human AF cells.

In previous studies, TGF-β1 was shown to increase collagen type I and II as well as GAG production in a rat foetal AF cell micromass model; additionally, it tended to promote cell proliferation (Hayes and Ralphs, 2011). Blanquer *et al*. (2017) reported that TGF-β3 is essential to support AF Differentiation of human adipose stem cells. However, the effects of TGF-β on the functional properties of AF cells and cell fate of MSCs were not clear, as markers of functional AF cells were not tested in those studies. The present study results corroborated the hypothesis that TGF-β1 induced a functional AF cell phenotype in human mildly degenerated AF cells, as indicated by upregulated expression of phenotypic markers (*CD146*, *SM22α* and *MKX*) and increased cell contractility. These results indicated that TGF-β1-induced AF cells from mildly degenerated disc tissue may be used as a cell source for AF rupture repair. It would be meaningful to further investigate the effect of TGF-β1 on AF phenotype Differentiation of MSCs under an appropriate microenvironment, which may expand the cell source options with an easily accessible endogenous cell type.

Interestingly, results showed that TGF-β1 induced *COL2A1* expression in human AF cells under *in vitro* 2D culture condition (Fig. 2a). However, in the *ex vivo* organ culture study, addition of TGF-β1 within the hydrogel induced *COL1A1* expression of human AF cells (Fig. 7), highlighting the context-specific behaviours of TGF-β1. These results indicated that the native AF tissue microenvironment *in situ* may orient the effect of TGF-β1 towards the native cell phenotype. *ELN* expression was also upregulated in implanted AF cells by TGF-β1 supplementation (Fig. 7), which plays an important role in maintenance of collagen organisation and recovery of the disc size and shape after deformation (Yu *et al.*, 2002). Nakai *et al.* (2016) showed that *ELN* upregulation in CD146^+^ AF cells may contribute to the development of a contractile phenotype in these AF cells. *SCX*, a basic helix-loop-helix transcription factor that marks the tendon/ligament cell lineage and is present in the AF (Pryce *et al.*, 2007; Yoshimoto *et al.*, 2017), was highly upregulated in both 2D and 3D cultures treated with TGF-β1 (Fig. 2a, 4). Interestingly, it had lower expression in oAF *vs*. NP in bovine IVD (Fig. 1). However, SCX^+^ AF cells function in healing of AF rupture in neonatal mice (Torre *et al.*, 2018), which may explain the low expression level in mature bovine AF tissue. IVD cells fail to maintain the balance of anabolism and catabolism during ageing and degeneration, with decreased ECM synthesis and increased ECM degrading enzymes, such as MMP and ADAMTS family. The upregulation of MMP3 and ADAMTS5 has been reported in degenerated IVDs (Vo *et al.*, 2013). Present results showed that TGF-β1 not only upregulated ECM production but also downregulated the expression of ECM catabolic enzymes MMP3 and ADAMTS5 (Fig. 2a, 4). This indicated that TGF-β1-treated AF cells may possess an anti-catabolic effect in AF rupture repair for prevention of further tissue degeneration. When AF cells were cultured in collagen I hydrogel both *in vitro* and *ex vivo*, higher cell density was found in TGF-β1-supplemented scaffolds (Fig. 3,6), which indicated that TGF-β1 promoted cell proliferation and/or cell survival of human AF cells. This is essential for rebuilding of the biological function when cells are implanted into the avascular disc milieu. In summary, results showed that TGF-β1 was an effective agent for biological functional recovery of AF.

PU materials are promising biomaterials for AF regeneration, as they are biodegradable, biocompatible, mechanically robust and elastic (Agnol *et al.*, 2018; Yeganegi *et al.*, 2010). In the present study, AF cells were evenly distributed within the porous PU scaffold, with or without collagen gel as a cell carrier (Fig. 3a–c). Furthermore, the scaffold size can be easily tuned for custom-designed patient need in further application. Type I collagen is a component of AF matrix and was used to deliver the functional AF cells in PU scaffold and create a biomimetic environment of AF. Collagen type I has been reported to promote AF cells proliferation and matrix production *in vitro* (Xiao *et al.*, 2017). Present results showed that collagen I hydrogel maintained AF cell survival after 7 d of 3D culture *in vitro* but did not further enhance cell proliferation. Importantly, collagen I hydrogel as a cell carrier could preserve the functional phenotype of TGF-β1-induced human AF cells, as indicated by higher expression level of *CD146*, *SM22α* and *MKX* as compared with PU scaffold only (Fig. 4). These results demonstrated the potential capacity of collagen I hydrogel for cell delivery and functional phenotype maintenance in AF rupture repair.

In the *ex vivo* preclinical study, AF defects were repaired with PU-Col constructs and PU membrane sealing. After 14 d of organ culture under daily dynamic loading, PU-Col constructs could still completely fill the AF defect and no herniation of NP tissue was found. While in injury control discs, part of the NP tissue protruded into the AF defect area (Fig. 6). Implantation of AF cells into the AF defect was carried out by delivering them in PU-Col constructs. After 14 d of *in situ* culture with daily dynamic load, the implanted AF cells remained in the PU-Col constructs and started matrix production. Interestingly, cell density and matrix production were more pronounced when TGF-β1 had been added in the constructs. On the other hand, cells expressed significantly higher *COL1A1* and showed a trend for higher levels of *CD146* and *ELN* in TGF-β1-supplied constructs. These results indicated that TGF-β1 administration within the selected biomaterial could better facilitate the enhancement of functional AF cell phenotype and neo-tissue formation *in situ*. Pirvu *et al.* (2015) showed that implantation of MSCs positively modulates cell phenotype of host disc tissue by up-regulating anabolic mechanisms and down-regulating catabolic mechanisms by paracrine effect. However, there was no evidence showing the same effect on native disc tissue with AF cells implantation (Fig. 8); while new matrix production was only observed with the functional AF cells implantation. These findings suggested that MSCs and AF cells had Different mechanisms of action. MSCs, which can be derived from a wide range of sources, can positively modulate cells of the host disc tissue, but may have limited potential for AF matrix production. In contrast, AF cells are suitable for neo-tissue formation, but do not influence the phenotype of adjacent host cells. To further improve the homeostasis in AF tissue repair and prevent further IVD degeneration, combination of functional AF cells and the effective paracrine factors from MSCs may bring further valued outcomes.

The limitations of the current study remain in the following aspects: the random porous architecture of the PU scaffold likely did not promote matrix deposition in an angle-plied lamellar microstructure similar to that of native AF fibres, despite its good cell seeding capacity. Implementation of additive manufacturing for production of PU scaffolds may further promote maintenance/induction of the functional AF cell phenotype and formation of a native-mimicking ECM structure. Both *in vitro* and *ex vivo* studies were performed with 2 weeks follow-up. Further longer follow-up study may be needed to evaluate long-term culture properties such as cell survival, ECM production and degradation of PU scaffold. Additional studies with high force, fatigue and complex loading to evaluate herniation risk of the construct are needed. Ultimately, *in vivo*, a higher load is expected in the disc. A press-fit and biocompatible material filler is needed to close the defect and restore the mechanical function of the IVD with high adhesion strength and adequate compressive, shear and tensile moduli (Long *et al.*, 2016), to provide structural support for the biological repair.

## Conclusions

The current study explored a novel AF repair strategy aiming at functional cell phenotype induction. A set of AF cell phenotype markers were defined in healthy AF tissue as compared with NP, including *COL1A2*, *CD146*, *SM22α* and *MKX*. TGF-β1 upregulated gene and protein expression of the AF cell markers in human mildly degenerated AF cells and increased cell contractility, indicating that TGF-β1-pre-treated AF cells may be an appropriate cell source for AF tissue engineering or rupture repair. Collagen type I hydrogel as a cell carrier in the PU scaffold maintained the phenotype of human AF cells. TGF-β1 treatment within the collagen hydrogel further promoted cell proliferation and matrix production of AF cells both *in vitro* and *ex vivo*. TGF-β1 and collagen type I hydrogel-PU scaffold hybrid system retained the AF phenotype of implanted cells. These constructs have potential for generating tissue engineered AF and warrant further investigation for their use in repairing AF defects after discectomy.

## Figures and Tables

**Table 1.** Oligonucleotide primers and probes (bovine and human) used for qRT-PCR. Primers and probes with the sequence shown were custom designed; primers and probes with the catalogue number were from Applied Biosystems. fw: forward; rev: reverse; b: bovine; h: human.

Gene	Primer/probe type	Sequence
***bCOL1A2***	Primer fw (5′−3′)	TGC AGT AAC TTC GTG CCT AGC A
	Primer rev (5′−3′)	CGC GTG GTC CTC TAT CTC CA
	Probe (5′FAM/3′TAMRA)	CAT GCC AAT CCT TAC AAG AGG CAA CTG C
***bCOL2A1***	Primer fw (5′−3′)	AAG AAA CAC ATC TGG TTT GGA GAA A
	Primer rev (5′−3′)	TGG GAG CCA GGT TGT CAT C
	Probe (5′FAM/3′TAMRA)	CAA CGG TGG CTT CCA CTT CAG CTA TGG
***bACAN***	Primer fw (5′−3′)	CCA ACG AAA CCT ATG ACG TGT ACT
	Primer rev (5′−3′)	GCA CTC GTT GGC TGC CTC
	Probe (5′FAM/3′TAMRA)	ATG TTG CAT AGA AGA CCT CGC CCT CCA T
***bMMP3***	Primer fw (5′−3′)	GGC TGC AAG GGA CAA GGA A
	Primer rev (5′−3′)	CAA ACT GTT TCG TAT CCT TTG CAA
	Probe (5′FAM/3′TAMRA)	CAC CAT GGA GCT TGT TCA GCA ATA TCT AGA AAA C
***bADAMTS5***	Primer fw (5′−3′)	GAT GGT CAC GGT AAC TGT TTG CT
	Primer rev (5′−3′)	GCC GGG ACA CAC CGA GTA C
	Probe (5′FAM/3′TAMRA)	AGG CCA GAC CTA CGA TGC CAG CC
***bCD146***		Bt03258894_m1
***bSM22α***		Bt03234600_m1
***bSCX***		Hs03054634_g1
***bMKX***		Bt04292311_m1
***bELN***		Bt03216594_m1
***bVCAN***		Bt03217632_m1
***bRPLP0***		Bt03218086_m1
***HCOL1A1***	Primer fw (5′−3′)	CCC TGG AAA GAA TGG AGA TGA T
	Primer rev (5′−3′)	ACT GAA ACC TCT GTG TCC CTT CA
	Probe (5′FAM/3′TAMRA)	CGG GCA ATC CTC GAG CAC CCT
***HCOL2A1***	Primer fw (5′−3′)	GGC AAT AGC AGG TTC ACG TAC A
	Primer rev (5′−3′)	GAT AAC AGT CTT GCC CCA CTT ACC
	Probe (5′FAM/3′TAMRA)	CCT GAA GGA TGG CTG CAC GAA ACA TAC
***hACAN***	Primer fw (5′−3′)	AGT CCT CAA GCC TCC TGT ACT CA
	Primer rev (5′−3′)	CGG GAA GTG GCG GTA ACA
	Probe (5′FAM/3′TAMRA)	CCG GAA TGG AAA CGT GAA TCA GAA TCA ACT
***hMMP3***		Hs00968305_m1
***hADAMTS5***		Hs01095518_m1
***hCD146***		Hs00174838_m1
***hSM22α***		Hs00162558_m1
***hSCX***		Hs03054634_g1
***hMKX***		Hs00543190_m1
***hELN***		Hs00355783_m1
***hVCAN***		Hs00171642_m1
***hRPLP0***	Primer fw (5′−3′)	TGG GCA AGA ACA CCA TGA TG
	Primer rev (5′−3′)	CGG ATA TGA GGC AGC AGT TTC
	Probe (5′FAM/3′TAMRA)	AGG GCA CCT GGA AAA CAA CCC AGC

## Discussion with Reviewers

**Cornelia Neidlinger-Wilke**: Does the organ culture model really simulate *in vivo* loading conditions? The risk of herniation is certainly lower under the applied experimental conditions. Could you please comment on this?

**Authors**: In the current study, the IVD organs were cultured under physiological axial compressive loading condition. This model partially simulated the *in vivo* loading conditions by resulting in a disc height loss of ~ 10 % after loading, which is comparable to the disc height loss of human lumbar IVD after normal daily activity. However, this model does not simulate the complex *in vivo* loading condition, including flexion, extension and torsion. In this respect, the risk of herniation is certainly lower under the applied experimental conditions. Further mechanical test under complex loading condition is needed to assess the biomechanical compatibility of the implanted constructs and fixation method. The current model simulated the biological microenvironment of *in vivo* native disc tissue for testing of the implanted constructs, representing a good pre-animal model to evaluate the biological compatibility of the implanted constructs in addition to an *in vitro* system.

**Cornelia Neidlinger-Wilke**: Regarding a possible *in vivo* application of such an approach, is this also an appropriate solution under the high loaded conditions in the physiological disc environment? Does the PU membrane sealing also persist under complex loading conditions? How can the risk of extrusion under high and complex load exposure be reduced?

**Authors**: We agree with the reviewer that many more challenges remain to be solved regarding the application of this method for AF repair *in vivo*. In the current study, EPIGLU® (Meyer-Haake GmbH) was used to stick a membrane for maintaining the scaffold in place under a loaded environment. In a previous study, a membrane-suture method was used to fix the implant (Pirvu *et al.*, 2015). These could all be possible options for scaffold fixation. For easier operation-room practice, the cell-seeded scaffold could be further developed into an adhesive patch to avoid manipulation hassles.

**Fackson Mwale**: In the NP, TGF-β1 downregulates CCL4 expression through ERK1/2 signalling activation (Zhang *et al.*, 2017, additional reference). What are the mechanisms underlying the effects of TGF-β1 on AF phenotypic induction?

**Authors**: According to Zhang *et al.* (2017), TGF-β1 can prevent degenerative processes, inhibit inflammatory responses in the dorsal root ganglia and prevent pain development, by inhibiting CCL4 expression *via* ERK1/2 signalling activation in NP cells. However, the mechanism of TGF-β1 on functional AF phenotypic induction is yet unclear. Preliminary results showed that ERK1/2 inhibitor promoted AF functional marker expression independent of TGF-β1 treatment, which indicated that ERK1/2 signalling pathway might also play a role in the regulation of AF phenotype markers expression. Ongoing study suggests that Smads pathway may play a key role in the effects of TGF-β1 on AF phenotypic induction. Further experiments are needed to confirm these preliminary findings.

**Fackson Mwale**: Would intradiscal injections of TGF-β1 and AF cells be effective?

**Authors**: Intradiscal injection of TGF-β1 and AF cells may be effective for repair of small AF ruptures. In case of a large AF defect, an adhesive and biocompatible material filler to close the defect and restore the mechanical function of the IVD will be needed to provide structural support for the biological repair.

**Fackson Mwale**: One of the limitations of the study, as pointed out, was the lack of the angle-ply, fibrillar organisation of the native AF. Would a 3D-printed scaffold in which the layers are printed in opposing angular orientations replicating the angle-ply arrangement of the native tissue be suitable for the collagen PU scaffold used?

**Authors**: It is a great suggestion to use a 3D-printed scaffold to mimic the angle-plied lamellae structure of the native AF tissue. Christiani *et al.* (2019, additional reference) developed a tissue engineered AF by 3D-printed polycaprolactone scaffolds with angle-plied architecture. It would be also suitable with collagen and PU, since both collagen I and PU have been widely used for 3D bioprinting. One potential direction is to print PU with an angle-plied structure as a backbone platform to supply the mechanical property and to print another layer of collagen I encapsulated with functional cells and bioactive agents within the space between the PU layers.

**Fackson Mwale**: How does this scaffold compare with the tensile, compressive and torsional mechanics of the AF?

**Authors**: The unconfined compressive stiffness of porous PU scaffold is approximately 20 kPa, which is weaker than the native AF tissue (0.19–0.26 MPa) (Freeman *et al.*, 2013, additional reference). The tensile and torsional mechanical properties of the PU scaffold were not measured in the current study. Further testing is needed to fully understand the mechanical property of the scaffold. The mechanical properties of the scaffold may be improved by mimicking the angle-plied lamellar microstructure of AF instead of random porous architecture. This may be achieved by using other technologies, such as electrospinning and 3D printing in further studies.

## List of Abbreviations

ACAN: aggrecan
ADAMTS5: a disintegrin and metalloproteinase with thrombospondin type I motifs, member 5
AF: annulus fibrosus
αMEM: alpha minimum essential medium
APC: allophycocyanin
BSA: bovine serum albumin
CCL4: hemokine (C-C motif) ligands 4
CD146: cluster of differentiation 146
COL1A2: collagen type I alpha 2
COL1A1: collagen type I alpha 1
COL2A1: collagen type II alpha 1
DAPI: 4′,6-diamidino-2-phenylindole
DMEM: Dulbecco′s modified Eagle medium
ECM: extracellular matrix
EDTA: ethylenediaminetetraacetic acid
ELN: elastin
EP: endplate
ERK: extracellular signal-regulated kinases
FACS: fluorescence-activated cell sorting
FAM: carboxyfluorescein
FBS: foetal bovine serum
FCS: foetal calf serum
GAG: glycosaminoglycan
HMDI: 1,6-hexamethylene diisocyanate
iAF: inner AF tissue
IVD: intervertebral disc
ISO: 1,4,3,6-dianhydro-D-sorbitol
LBP: low-back pain
MMP3: matrix metalloproteinase 3
MKX: mohawk homeobox
MSCs: mesenchymal stem cells
MRI: magnetic resonance imaging
NEAA: non-essential amino acid
NP: nucleus pulposus
oAF: outer AF tissue
PBS: phosphate-buffered saline
PCL: poly(ε-caprolactone)
PU: polyurethane
PU-Col: PU scaffold-collagen hydrogel
PU-Col-AFCs: PU-Col with TGF-β1 pre-treated AF cells
PU-Col-AFCs-TGFβ: PU-Col-AFCs and 5 ng TGF-β1
qRT-PCR: quantitative real-time polymerase chain reaction
RPLP0: ribosomal protein lateral stalk subunit P0
SCX: scleraxis
SM22α: smooth muscle protein 22-alpha
TAMRA: tetramethylrhodamine
TGF-β1: transforming growth factor β1
VCAN: versican
